# Rabies virus-neutralising antibodies in healthy, unvaccinated individuals: What do they mean for rabies epidemiology?

**DOI:** 10.1371/journal.pntd.0007933

**Published:** 2020-02-13

**Authors:** Susannah Gold, Christl A. Donnelly, Pierre Nouvellet, Rosie Woodroffe

**Affiliations:** 1 Institute of Zoology, Zoological Society of London, London, United Kingdom; 2 MRC Centre for Global Infectious Disease Analysis, Department of Infectious Disease Epidemiology, School of Public Health, Imperial College London, London, United Kingdom; 3 Department of Statistics, University of Oxford, Oxford, United Kingdom; 4 School of Life Sciences, University of Sussex, Falmer, United Kingdom; Centers for Disease Control and Prevention, UNITED STATES

## Abstract

Rabies has been a widely feared disease for thousands of years, with records of rabid dogs as early as ancient Egyptian and Mesopotamian texts. The reputation of rabies as being inevitably fatal, together with its ability to affect all mammalian species, contributes to the fear surrounding this disease. However, the widely held view that exposure to the rabies virus is always fatal has been repeatedly challenged. Although survival following clinical infection in humans has only been recorded on a handful of occasions, a number of studies have reported detection of rabies-specific antibodies in the sera of humans, domestic animals, and wildlife that are apparently healthy and unvaccinated. These ‘seropositive’ individuals provide possible evidence of exposure to the rabies virus that has not led to fatal disease. However, the variability in methods of detecting these antibodies and the difficulties of interpreting serology tests have contributed to an unclear picture of their importance. In this review, we consider the evidence for rabies-specific antibodies in healthy, unvaccinated individuals as indicators of nonlethal rabies exposure and the potential implications of this for rabies epidemiology. Our findings indicate that whilst there is substantial evidence that nonlethal rabies exposure does occur, serology studies that do not use appropriate controls and cutoffs are unlikely to provide an accurate estimate of the true prevalence of nonlethal rabies exposure.

## Introduction

Rabies virus (RABV) is a negative-sense RNA virus in the Rhabdoviridae family and one of 16 currently described viruses in the *Lyssavirus* genus [1]. Whilst other lyssaviruses also cause fatal disease that is indistinguishable from that caused by RABV, RABV is the greatest threat to human health. Typically transmitted in the saliva of infected hosts through bites, it is highly neurotropic and causes mortality through encephalomyelitis. Rabies kills an estimated 59,000 people annually, many of whom are children [2]. In rural Africa and Asia, where the majority of human cases occur, domestic dogs are the primary reservoir, responsible for up to 99% of rabies transmission to humans [3].

The ecology of rabies is complex, with transmission occurring between wildlife, domestic animals, and humans. However, as the host species responsible for the majority of transmission to humans, domestic dogs are the primary target for vaccination [4]. The World Health Organization (WHO) recommends vaccination coverage of at least 70% of the domestic dog population, repeated over several annual campaigns, to achieve rabies control [3]. This coverage level is supported by mathematical models of rabies dynamics [5,6,7]. However, rabies epidemiology is highly variable, with differences in viral strain, domestic dog density, and wildlife involvement between locations [8,9]. One area of this complex ecology of rabies that has long been recognised, but the significance of which remains unclear, is the occurrence of rabies virus-neutralising antibodies (RVNAs) in healthy, unvaccinated individuals.

## Methodology

We conducted multiple searches using electronic databases, including Web of Science and Google Scholar. The search strategy involved using different combinations of the following words and phrases to identify relevant publications: rabies, lyssavirus, serology, antibodies, nonlethal, nonfatal, carrier, latent, recovery, survival, and ‘rabies virus-neutralising antibodies’. The search covered all years and any studies in the English language. We also searched reference lists from articles identified for other relevant sources. We selected studies that were relevant under the following categories: 1) rabies serology studies in unvaccinated humans, domestic dogs, and wildlife; 2) reviews and studies on rabies serology tests; 3) studies on nonlethal rabies exposure, both experimental and under field conditions; and 4) studies on rabies mathematical modelling and surveillance with relevance to implications of naturally acquired immunity. The serology studies included in Tables 1 and 2 are not an exhaustive list but were selected as examples to cover a range of species, countries, and test methods.

**Table 1:** Serology surveys in unvaccinated domestic dogs yielding the estimated percentage with detectable rabies-specific antibodies in serum.

Country	Seroprevalence (95% CI)	Sample Size (# Positive)	Test Method	Cutoff	Seropositive Titre Range	Ref
**Nigeria**	16.1 (11.8–21.3)	254 (41)	HI	1:16	1:16–1:1,024	[114]
**Nigeria**	30.7 (26.5–35.1)	463 (142)	RFFIT; MNT	1:8	1:8–1:256	[11]
**Ethiopia**	80.0 (44.3–97.5)	10 (8)	RFFIT; ELISAs	RFFIT: 1:50 ELISA: 0.5 IU/0.2 mL	RFFIT: 1:60–1:540 ELISA: 1.2–1.8 IU/mL	[35]
**Kenya**	9.6 (5.7–14.9)	178 (17)	Modified RFFIT	0.5 IU/mL	NA	[115]
**Namibia**	30.0 (19.6–42.1)	70 (21)	LPBE	>log_10_1.4	log_10_1.4–log_10_2.8	[53]
**Tanzania**	LPBE: 7.4 (5.4–9.9) RFFIT: 49.4 (42.7–56.0)	567 (42) 233 (115)	RFFIT; LPBE	RFFIT: 0.5 IU/mL ELISA: >log_10_1.5	RFFIT: 0.5–2.7 IU/mL ELISA: log_10_1.5–log_10_1.8	[33]
**Tunisia**	LPBE: 28.8 (20.9–37.9) RFFIT: 39.0 (30.1–48.4)	118 (34) 118 (46)	RFFIT; ELISA	RFFIT: 0.5 IU/mL ELISA: 0.5 EU/ml	NA	[30]
**China**	13.3 (1.7–40.5)	15 (2)	RFFIT	0.5 IU/mL	NA	[94]
**Kenya**	28.0 (18.2–39.6)	75 (21)	RFFIT	0.05 IU/mL	0.05 to >0.56 IU/mL	[51]
**Uganda**	19.8 **(**12.5–28.9)	101 (20)	FAVN	0.24 IU/mL	NA	[116]

The 95% CI for the percentage seropositivity is shown. Sample size is shown with the number of seropositive individuals reported in brackets. Type of serology test and cutoff threshold used to define a seropositive are shown. When provided, the cutoff titre is shown converted to IU; otherwise, the dilution is shown. When reported, the range of titres in individuals defined as seropositive is shown; otherwise, this is recorded as NA. Further information on each study is provided in S1 Table in the supporting information. **Abbreviations:** CI, confidence interval; ELISA, enzyme-linked immunosorbent assay; EU, equivalent units; FAVN, fluorescent antibody virus neutralisation; HI, haemagglutinin inhibition; IU, international units; LPBE, liquid-phase–blocking ELISA; MNT, mouse neutralisation test; NA, not available; RFFIT, rapid fluorescent focus inhibition test.

**Table 2:** Serology surveys in unvaccinated wildlife yielding the estimated percentage with detectable rabies-specific antibodies in serum.

Species	Country	Seroprevalence (95% CI)	Sample Size (# Positive)	Cutoff	Test Method	Seropositive Titre Range	Ref
**Black-backed jackal** (*Canis mesomelas*)	Kenya	3.6 (0.1–18.4)	28 (1)	0.5 IU/mL	RFFIT	NA	[117]
	Kenya	1.4 (0.0–7.8)	69 (1)	0.05 IU/mL	RFFIT	0.25 IU/mL	[51]
	Namibia	8.6 (3.6–17.0)	81 (7)	1:10	FAVN	NA	[74]
**African wild dog** (*Lycaon pictus*)	Tanzania	25.0 (5.5–57.2)	12 (3)	0.5 IU/mL	RFFIT	All 0.55 IU/mL	[54]
	Kenya	8.6 (2.9–19.0)	58 (5)	0.05 IU/mL	RFFIT	0.067 to >0.418 IU/mL	[51]
**Wolf** (*Canis lupus*)	US	1.1 (0.0–6.2)	88 (1)	1:10	RFFIT	NA	[118]
**Ethiopian wolf** (*Canis simensis*)	Ethiopia	13.3 (1.7–40.5)	15 (2)	RFFIT: 1:50 ELISA: 0.5 IU/0.2 mL	RFFIT; ELISAs	RFFIT: both 1:60 ELISA: 1.2–2.5	[35]
**Arctic fox** (*Vulpes lagopus*)	US	4.3 (1.2–10.8)	92 (4)	1:5	Modified SNT	1:11–1:45	[97]
**Crab-eating fox** (*Cerdocyon thous*)	Brazil	5.9 (1.6–14.2)	68 (4)	0.10 IU/mL	Modified SNT	0.10–0.27 IU/mL	[73]
**Bush dog** (*Speothos venaticus*)	Brazil	100.0 (2.5–100.0)	1 (1)	0.10 IU/mL	Modified SNT	1.6 IU/mL	[73]
**Maned wolf** (*Chrysocyon brachyurus*)	Brazil	14.3 (7.8–23.2)	91 (13)	0.10 IU/mL	Modified SNT	0.1–0.27 IU/mL	[73]
**Spotted hyena** (*Crocuta crocuta*)	Tanzania	37.0 (27.6–47.2)	100 (37)	0.5 IU/mL	RFFIT	NA	[119]
	Kenya	6.5 (2.7–13.0)	107 (7)	0.05 IU/mL	RFFIT	0.09–0.29 IU/mL	[51]
**Small Indian mongoose** (*Herpestes javanicus*)	Puerto Rico	39.3 (30.2–49.0)	112 (44)	0.1 IU/mL	RFFIT	0.1–50.0 IU/mL	[98]
**Small Asian mongoose** (*Herpestes auropunctatus*)	Grenada	18.9 (16.0–22.1)	672 (127)	1:2	Modified SNT	1:5,900	[120]
**Pampas cat** (*Leopardus colocolo*)	Brazil	20.0 (0.5–71.6)	5 (1)	0.10 IU/mL	Modified SNT	0.13 IU/mL	[73]
**Jaguar** (*Panthera once*)	Brazil	23.1 (5.0–53.8)	13 (3)	0.10 IU/mL	Modified SNT	0.10–0.13 IU/mL	[73]
**Ocelot** (*Leopardus pardalis*)	Brazil	20.0 (2.5–55.6)	10 (2)	0.10 IU/mL	Modified SNT	0.10–0.13 IU/mL	[73]
**Puma** (*Puma concolor*)	Brazil	12.5 (0.3–52.7)	8 (1)	0.10 IU/mL	Modified SNT	0.10 IU/mL	[73]
**Lion** (*Panthera leo*)	Zambia	40.0 (19.1–64.0)	20 (8)	0.2 IU/mL	RFFIT	0.2–1.8 IU/mL	[45]
**Oncilla** (*Leopardus tigrinus*)	Bolivia	100 (2.5–100.0)	1 (1)	NA	RFFIT	>70 IU/mL	[55]
**Common Vampire Bat** (*Desmodus rotundus*)	Argentina	Pre: 3.0 (1.9–4.6) During: 6.6 (2.2–14.7) Post: 16.8 (10.3–25.3)	Pre: 694 (21) During: 76 (5) Post: 107 (18)	1:5	MNT	NA	[22]
	Brazil	7.4 (4.2–11.8)	204 (15)	0.5 IU/mL	ELISA; RFFIT	NA	[121]
**Big brown bat** *(Eptesicus fuscus)*	US	9.6 (5.8–14.8)	187 (18)	1:8	Modified SNT	NA	[122]
**Little brown bat** *(Myotis lucifugus)*	US	2.4 (0.5–6.8)	127 (3)	1:8	Modified SNT	NA	[122]
**Mexican free-tailed bat** (*Tadarida brasiliensis*)	US	68.5 (65.1–71.8)	750 (514)	1:10	Modified SNT	NA	[123]
**Human** (*Homo sapien*)	Peru	11.1 (4.6–21.6)	63 (7)	0.1 IU/mL	RFFIT	0.1–2.8 IU/mL	[13]
	Nigeria	28.6 (23.9–33.6)	350 (100)	1:8	RFFIT	1:8–1:64	[11]
**Capuchin monkey** (*Cebus paella*)	Brazil	6.7 (0.2–32.0)	15 (1)	0.11 IU/mL	RFFIT	0.33 IU/mL	[57]
	Brazil	11.1 (3.1–26.1)	36 (4)	0.25 IU/mL	RFFIT	0.7–1.3 IU/mL	[58]
**Black bear** (*Ursus americanus*)	US	5.2 (1.4–12.8)	77 (4)	1:5	RFFIT	1:20–1:320	[124]
**Opossum** (*Didelphis aurita*)	Brazil	11.0 (5.8–18.4)	109 (12)	0.11 IU/mL	RFFIT	0.11–1.00 IU/mL	[61]
**Striped skunk** (*Mephitis mephitis*)	US	9.1 (5.5–14.0)	198 (18)	0.09 IU/mL	RFFIT	0.13–2.36 IU/mL	[125]
**Raccoon** (*Procyon lotor*)	US	40.5 (24.8–57.9)	37 (15)	0.05 IU/mL	RFFIT	0.05 to <0.12 IU/mL	[56]
	US	MNT: 17.2 (13.1–22.0) RFFIT:25.3 (20.1–31.1)	297 (51) 253 (64)	1:2 1:5	MNT; RFFIT	1:5–1:125	[23]
**Crab-eating raccoon** (*Procyon cancrivorus*)	Brazil	7.7 (0.2–36.0)	13 (1)	0.10 IU/mL	Modified SNT	0.27 IU/mL	[73]
**Coati** (*Nasua nasua*)	Brazil	100.0 (15.8–100.0)	2 (2)	0.11 IU/mL	RFFIT	0.12–0.20 IU/mL	[57]

This is not an exhaustive list of all studies but covers a breadth of species and locations. The 95% CI for the percentage seropositivity is shown. Sample size is shown with number of seropositive individuals in brackets. The type of serology test and cutoff used are shown. A number of studies used SNTs other than the RFFIT or FAVN; for simplicity, these are referred to as modified SNTs. Where provided, the cutoff titre used to define a seropositive is shown converted to IU; otherwise, the dilution is shown. Pre, during, and post refer to time of sampling relative to a rabies outbreak in cattle in [22]. Further information on each study is included in S2 Table in the supporting information. **Abbreviations:** CI, confidence interval; ELISA, enzyme-linked immunosorbent assay; FAVN, fluorescent antibody virus neutralisation; IU, international units; MNT, mouse neutralisation test; NA, not available; RFFIT, rapid fluorescent focus inhibition test; SNT, serum neutralisation test.

## Studies of rabies-specific serum antibodies in healthy, unvaccinated individuals

Prior to the development of postexposure prophylaxis (PEP), exposure to rabies in humans was widely considered to be uniformly fatal. Although when promptly administered, PEP is highly effective in preventing clinical infection, following the onset of symptoms, the disease fatality rate is still effectively 100%. However, the inevitable fatality of untreated rabies exposure in humans has been challenged by several studies showing the presence of RVNAs in serum from apparently healthy humans who have not been vaccinated [10,11,12,13]. For example, in a study of two communities in Peru, 7 of 63 individuals tested were found to have detectable RVNAs, of which only one had any history of prior vaccination [13]. When a host is exposed to a pathogen, antibodies able to bind to the specific antigens present on the pathogen are selected for and amplified. If a host does not succumb to the exposure, this heightened response can be detected after the pathogen is cleared. Detectable levels of rabies-specific antibodies in healthy, unvaccinated individuals therefore suggest exposure to RABV that has not led to fatal disease. Whilst natural development of rabies antibodies in healthy individuals has rarely been recorded in humans, detectable RVNAs in sera have been reported in a number of studies in populations of domestic dogs and wildlife in regions where rabies is endemic (Tables 1 and 2).

Despite nonlethal rabies exposure first being reported in laboratory experiments by Pasteur in 1882 [14], whether detection of RVNAs in unvaccinated domestic dogs and wildlife is the result of nonlethal rabies exposure remains controversial. In bats, which have a long history of coevolution with lyssaviruses, it is well established that nonlethal rabies exposure regularly occurs [15]. However, whether this is true for other mammals is unclear. Interpretation of serology studies is complicated by variation in specificity of the test methods and cutoffs used and by potential cross-reactivity [16]. This has led to an unclear picture of the significance of nonlethal exposure for rabies epidemiology and control. Substantial work and discussion of this topic has previously been carried out by Bell, Carey and McLean, Fekadu, and Cleaveland and Dye [17,18,19,20].

## Detection methods for rabies antibodies

Several test methods are available for detecting rabies-specific antibodies. The first developed was the mouse neutralisation test (MNT). In this method, sera at different concentrations are mixed with a constant viral dose and are then inoculated into weanling mice. The mice are then observed for development of clinical symptoms [21]. This method was used in some early wildlife serology surveys (e.g., [22,23]) but has since been replaced by in vitro methods. The most commonly used in vitro methods are virus neutralisation tests and ELISAs (enzyme-linked immunosorbent assays).

Virus neutralisation tests, which include the rapid fluorescent focus inhibition test (RFFIT) and fluorescent antibody virus neutralisation (FAVN), are the approved methods for measuring vaccination response prior to movement of animals internationally [24]. Both the FAVN and RFFIT rely on the same principle, in which the antibody concentration is measured by the ability of sera to neutralise a challenge virus, detected by the reduction in fluorescence in virus-infected cells [25]. The difference between the two tests is the method of plate-reading. Several modifications of the standard virus neutralisation tests have also been developed, for example, to allow the use of small serum volumes and pseudotype viruses [26,27].

An alternative to neutralisation tests is the use of ELISAs. In contrast to the RFFIT and FAVN, ELISAs do not measure neutralisation, but estimate the antibody concentration in a serum sample able to bind specifically to rabies antigens. This concentration does not provide a direct measure of protection against the virus because the recorded titre also includes antibodies that bind but may not contribute to defence against the virus [28]. ELISAs are faster to run than the RFFIT and do not require live RABV or cell-culture facilities. A number of different ELISAs have been developed and are available commercially, but these have been shown to vary in sensitivity and specificity [29].

## Challenges to interpreting rabies serology tests

### Discordance between test methods

Both rabies neutralisation tests and ELISAs have primarily been used for measuring responses to vaccination. Comparison of these tests shows that for vaccinated domestic dogs, there is a strong correlation between the titres measured [30, 31]. However, in wild carnivores, the correlation following oral vaccination has been found to be poor, with challenge experiments suggesting that the ELISA potentially provides a better measure of protection [32]. In unvaccinated individuals, both neutralisation tests and ELISAs have been used to detect antibodies arising from natural exposure. However, when results from different tests have been compared, poor agreement has been found. In a sample of 286 dogs, Bahloul and colleagues found concordance between the two tests for only 30% of the dogs defined as unvaccinated seropositives [30]. Substantial discordance was also found by Cleaveland and colleagues, with significantly higher seroprevalence detected by RFFIT than ELISA [33].

The lack of agreement between rabies serology tests in unvaccinated individuals suggests these methods differ in their specificity or sensitivity for detecting nonlethal exposures. Cleaveland and colleagues found that an RFFIT, but not an ELISA, detected false positives on a rabies-free island, suggesting that using an ELISA may be more specific for detecting nonlethal exposures [33]. This lack of specificity with the RFFIT may be a result of non-antibody neutralising factors present in samples. If serum samples are of poor quality, cytotoxicity can also occur in neutralisation tests, resulting in false positives [34]. The discrepancies observed may also be partially explained by differences in the class of antibody detected by the two tests [33,35]. Neutralisation tests detect all classes of antibodies, whereas ELISAs specifically test for a single class of antibody [36]. Because different antibody classes are produced at different times following exposure, the two tests may differ in sensitivity depending on the time since exposure or vaccination.

### Defining cutoffs

For both virus neutralisation tests and ELISAs, distinguishing between seropositive and seronegative individuals requires defining a cutoff point. The higher a cutoff is set, the lower the probability that false positives are detected—however, the higher the chance of missing evidence of exposure. Typically, for the RFFIT in domestic dogs, a titre of 0.5 international units (IU)/mL is used, which is the threshold set by WHO as confirmation of antibody generated against vaccine [37]. This titre has also been shown to be protective in some wildlife species following vaccination, with individuals with a titre of >0.5 IU/mL having a >95% probability of survival following challenge [32]. As a result, in serology studies of unvaccinated wildlife and domestic dogs, this cutoff is often used as evidence as exposure (see Tables 1 and 2). However, whilst this cutoff has been validated in response to vaccination in dogs and a small handful of wildlife species, it has not been verified for detection of exposures in unvaccinated individuals. A number of serology studies have also used lower cutoffs as evidence of exposure, often without statistical justification. Methods are available to estimate the appropriate cutoff, such as receiver operating characteristic (ROC) curve analysis [38]. However, these methods rely on having known positive and negative controls for a species, and because of the difficulties of this for most wildlife species, few serology surveys employ these [39, 40]. A further challenge of interpreting rabies serology studies is that many do not convert to IU by comparison to a standard. Without standardisation, dilutions cannot be readily compared between studies.

### Cross-reactivity

A further factor that complicates interpretation of rabies serology tests is the possibility of cross-reactivity. For closely related viruses, similarity of antigens can allow antibodies generated against one virus to neutralise others. RVNAs could therefore indicate current infection or previous exposure to other lyssaviruses. However, the lyssaviruses are divided into 3 phylogroups based on serologic cross-reactivity and genetic distances within the G-protein codomain, and cross-reactivity is limited between phylogroups [41,42,43,44]. Whether RVNAs can be attributed specifically to exposure to RABV should therefore be considered within the specific context. Taking the example of lyssaviruses in sub-Saharan Africa: RABV is in phylogroup 1, whilst, with the exception of Duvenhage virus, which has only rarely been isolated, the majority of other lyssaviruses known to circulate are in phylogroup 2 or 3. By contrast, in Europe a number of other phylogroup 1 lyssaviruses circulate in bat species [1,41]. However, surveillance of lyssaviruses remains limited, and the current understanding of lyssavirus diversity and distribution may change with further study. In species that are known rabies reservoirs or that are likely to come into contact with these species, it may be justified to assume that exposure to RABV is more likely than exposure to other lyssaviruses. For example, Berentsen and colleagues found high seroprevalence of RVNAs in lions (*Panthera leo*), which, based on the species ecology, they considered to be more likely to have resulted from contact with canine rabies than lyssaviruses in bat populations [45]. When cross-reactivity is likely, serology tests using other lyssaviruses as challenge viruses could elucidate whether cross-reactivity is occurring [46]. For example, Ogunkoya and colleagues tested dog and human samples for neutralisation of RABV, Lagos virus, and Mokola virus and found no evidence of cross-reactivity [11]. In bat species, Mélade and colleagues also found no cross-reactivity in individuals seropositive for Duvenhage virus or European Bat Lyssavirus 1 (EBLV-1) and RABV, despite these belonging to the same phylogroup [47]. However, in foxes and racoon dogs vaccinated against RABV, strong cross-reactivity with both EBLV-1 and 2 has been shown [48]. As well as cross-reactivity with lyssaviruses, in human clinical cases, the indirect fluorescent antibody test has been found to detect false rabies seropositives in patients with encephalitis caused by West Nile and Powassan flaviviruses [49]. Therefore, antibodies in unhealthy individuals may not be considered specific. However, standard rabies neutralisation tests in this study detected no false positives, suggesting this is method-specific.

### Factors affecting sensitivity

As well as factors affecting the specificity of rabies serology tests, a number of factors can affect the sensitivity for detecting prior exposure. Repeated freezing and thawing of serum samples can cause antibodies to decay, reducing the sensitivity of serology tests [50,51]. Furthermore, the antibody level in an individual is affected by multiple factors, such as time since exposure and individual variation in immune response [16,52]. As a result, undetectable levels of antibody are not necessarily a true indicator that an individual has never been exposed because antibody levels may have decayed below the detectable threshold. For example, this has been observed in experimental rabies infection in big brown bats (*Eptesicus fuscus*) [15].

## Can RVNAs be attributed to nonlethal rabies exposure?

Given the challenges of rabies serology discussed so far, it is important to consider whether rabies seropositives reported in serology surveys can be attributed specifically to nonlethal exposure to RABV. Potentially the greatest challenge is whether the test methods used are generating false positives. The majority of the domestic dog studies reporting detection of rabies-specific antibodies used an RFFIT, which, as shown by Cleaveland and colleagues, may generate false positives, with 10.3% seroprevalence found using a 0.5 IU/mL cutoff on a rabies-free island [33]. This study provides significant evidence that at least a proportion of seropositives detected by RFFIT are likely to be false positives due to either nonspecific neutralisation or cross-reactivity. However, the same study found that using an ELISA, no false positives were detected, but 7.4% of dogs in a rabies-endemic region were seropositive. The location of these dogs also correlated with known rabies cases, providing strong evidence that these are true evidence of nonlethal exposures. Other studies using ELISAs in domestic dogs have also found high seroprevalences using similar cutoffs [30,53]. Therefore, whilst false positives are likely to occur, at least a proportion of seropositives in domestic dogs appear to be true indicators of prior nonlethal exposure.

The lack of specificity of the RFFIT in domestic dogs, for which the test has been validated, raises serious concerns for its use in wildlife species, for the majority of which little or no validation of the serology tests has been conducted. As with domestic dog studies, in most of the wildlife studies identified, the RFFIT was used (Table 2). In almost no cases was a comparison to a rabies-free control population made. Exceptions were Gascoyne and colleagues and Deem and colleagues, who used zoo populations, although in both cases only for very small sample sizes [54,55]. A majority of the studies made no mention of what controls were used or used domestic dog or human controls. A number of wildlife studies also used cutoffs lower than 0.5 IU/mL, which increases the probability of detection of false positives. In a number of the studies considered, if the cutoff had been 0.5 IU/mL, no seropositives would have been detected (e.g., [56,57]). However, in other cases, the antibody titres found were well above the standard cutoff point (see Table 2). For example, Machado and colleagues detected titres of up to 1.3 IU/mL in capuchin monkeys (*Cebus apella nigritus*), Berentsen and colleagues up to 1.8 IU/mL in lions, and Deem and colleagues a titre of greater than 70 IU/mL in an oncilla (*Leopardus tigrinus*) [45,55,58]. However, given that the serology tests have not been validated for these species, interpretation of these high titres is unclear. A further challenge for interpreting wildlife serology studies is that in a number of cases, the sample sizes used were small, leading to wide confidence intervals for the seroprevalence estimates. Verifying serology methods for the target species and achieving larger sample sizes should therefore be a priority for rabies serology surveys in wildlife.

In domestic dog studies, whilst the serology tests have been verified for this species, interpreting serology studies is complicated by the potential that seropositive individuals have been vaccinated, but this has not been recognised because of poor record keeping. Whilst this is possible in some cases, the majority of studies reported in Table 1 were conducted on owned dogs and in areas where little or no vaccination had been carried out. It is therefore presumed that the owners or researchers will know whether dogs had been previously vaccinated with relative surety. Whilst it remains possible that a proportion of individuals were vaccinated, it appears unlikely that vaccination would account for a significant proportion of the seropositives detected. In areas where oral vaccination of wildlife has been carried out, it is also possible that this could explain seropositivity. However, the wildlife studies reported were primarily carried out in areas with no wildlife vaccination or prior to distribution of vaccine (e.g., [53,59]).

Overall, rabies serology studies should be interpreted critically, and reported seroprevalences are unlikely to provide a completely accurate estimate of nonlethal exposure. However, it appears that at least a proportion of seropositives are likely to indicate true incidents of rabies exposures. Studies using rabies serology to infer exposure to the virus would benefit from greater consideration of the most suitable test method and cutoff to provide high specificity and of the use of species-specific controls.

## Alternative courses of rabies infection as the cause of RVNAs

In ‘classical’ rabies infection, following the latent period, clinical infection is short-lived. Infected individuals display furious or paralytic symptoms, followed by death from encephalomyelitis. Typically, because of immune evasion by the virus, neutralising antibody responses are only detectable at a late stage in the course of clinical infection, by which point the infected individual is unable to effectively clear the virus [60]. For example, a study of human clinical cases found that rabies antibodies in serum were not detected until around 10 days after the onset of clinical symptoms [61]. Occurrence of rabies-specific antibodies in healthy, unvaccinated individuals must therefore be the result of an alternative course of rabies exposure. Four alternative courses of rabies infection that could lead to rabies antibody detection in healthy individuals were initially identified by Fekadu [19]. These are subclinical infection, recovery from clinical infection, a carrier state, and an extended latent period [19].

### Subclinical infection

Subclinical infection refers to the clearance of the virus before the onset of recognisable clinical symptoms. As shown by the effectiveness of vaccination, rabies antigens are highly immunogenic [60]. In classical rabies infection, immune evasion by the virus in the central nervous system (CNS) results in a failure of the host to develop an effective antibody response [62]. However, if clearance of the virus can be achieved before entry into the CNS, clinical infection can be avoided, leading to a subclinical or ‘aborted’ infection with mild or no symptoms. A number of studies have observed subclinical infections following experimental exposure with RABV, including in dogs [63,64] and mice [65,66]. In bats, the occurrence of subclinical rabies infection is particularly prevalent [67]. Several studies have demonstrated survival of bats following challenge with RABV without development of symptoms and in some cases with subsequent development of RVNAs [15,67,68,69,70]. Lyssaviruses have coevolved with bat species, which, though susceptible to lyssaviruses, have considerably lower fatality rates when exposed compared to other mammals [71,72]. In other mammals, in cases in which true rabies-specific antibodies are detected in healthy, unvaccinated individuals under field conditions, subclinical exposure also appears to be the most parsimonious explanation. Seropositive individuals have been shown to have no evidence of symptoms, and later development of clinical infection has not been observed [33,73,74]. In the situation of recovery or an extended latent period, evidence of previous clinical symptoms or lower survivorship due to later development of rabies would be expected.

The probability of developing clinical rabies infection, relative to subclinical, will vary depending on the host species and viral stain. In vampire bats, Blackwood and colleagues estimated the probability of developing fatal infection following exposure to be 0.1 [75]. By contrast, in a study of domestic dogs, the proportion of exposed dogs that developed clinical infection was considerably higher at 0.49 (95% confidence interval: 0.45–0.52) [76]. In humans who did not receive postexposure treatment after a probable rabies exposure, Changalucha and colleagues found that 0.165 developed rabies [77]. This probability was dependent on the site of exposure, with the highest risk from bites to the head [77]. Of these exposures not leading to clinical infection, it may be that the reported contacts were not with infectious individuals or that contact failed to lead to viral establishment, with the host remaining susceptible. However, a proportion of these exposures may be cleared by the immune system and lead to development of immunity. Although further study is needed, subclinical infection appears to be the most likely alternative course of rabies infection that could lead to RVNA development under natural conditions.

### Recovery

Recovery here means clearance of the virus following the onset of recognisable clinical symptoms, in contrast to subclinical infection, in which clearance occurs before these symptoms appear. Recovery from clinical infection in domestic dogs was first reported by Pasteur in 1882, who noted that dogs can ‘sicken and recover’ from rabies [14]. Recovery in humans has also been reported on a handful of occasions; however, in most cases only when vaccine had been administered before onset of clinical symptoms [78]. When recovery has occurred, the majority of cases were left with permanent neurologic sequelae and severe cognitive disability [79]. In experimental infection of animals, recovery has been recorded in a number of species, including mice [17,66,80], dogs [81], a ferret [82], and rabbits [83]. As with human cases, the majority of these individuals had lasting sequelae following recovery.

Symptomatic rabies infection occurs after the entry of the virus of the CNS. Therefore, recovery depends on clearance of the virus from the CNS. However, once the virus is in the CNS, it is protected from the humoral immune response unless antibody is able to cross the blood–brain barrier (BBB). In cases in which recovery has been recorded, permeability of the BBB and the presence of antibody in the cerebrospinal fluid (CSF) have been shown to be key correlates of recovery [63,84,85,86]. As a result, in cases in which antibody is detected in the CSF of healthy individuals as well as the serum, this provides a key indication of recovery rather than subclinical infection. Because parenteral vaccination only leads to development of serum antibody, CSF antibody can also rule out that vaccination has occurred [87].

Whilst recovery can occur, in most cases, development of CSF antibody occurs too late to allow clearance [60]. Reports of recovery under natural conditions are scarce, despite the distinctive clinical symptoms associated with rabies that are likely to increase the probability of detecting cases of recovery relative to subclinical infection. For example, in a study of 957 naturally infected dogs, none survived more than 10 days after admission into quarantine [88]. In the case of wildlife, survival with clinical symptoms may be even less likely as because of the debilitating nature of the disease, they would be highly vulnerable to predation or starvation. Therefore, given the scarcity of recorded cases of natural rabies recovery and the permanent disability associated with clinical rabies, it is unlikely to be a common course following rabies exposure under field conditions. However, testing for antibodies in the CSF of individuals with seropositive sera could allow for distinction between subclinical infection and recovery.

### Carrier state

In the carrier state, the infected host sheds virus across an extended period whilst remaining apparently clinically healthy. The occurrence of the carrier state for rabies is highly controversial. The first suggestion of its occurrence was in vampire bats (*Desmodus rotundus*) [89]; however, later experimental studies testing for carriers in this species found little evidence, with excretion in the saliva only detected for a short period after clinical infection [90,91]. Potentially, a greater cause for concern is if domestic dogs are able to act as carriers, in which case bites from apparently healthy individuals could still cause human deaths. A carrier state has occasionally been reported in domestic dogs; for example, in one experimentally infected dog, RABV was isolated from saliva up to 305 days following recovery from clinical infection until death during whelping [92]. In Nigeria, Aghomo and Rupprecht isolated RABV from the saliva of 4 healthy, unvaccinated dogs brought for routine veterinary examination [93]. The strains isolated were found to be pathogenic in mice, but not in puppies, which was taken as evidence for a host-adapted strain with lower pathogenicity. A further study of 153 dogs in China found rabies antigen in the saliva of 15 dogs. However, observation of these dogs for 6 months showed detection of antigen was inconsistent, and no viral RNA was detected, suggesting the ELISA may have provided unreliable results [94]. Two further studies screening dogs in Argentina and India found no evidence for a carrier state [95,96]. Therefore, clear evidence for a carrier state in domestic dogs is very limited. In wildlife, evidence for a carrier state is also scarce. Of the wildlife serology studies considered, a number tested for virus present in the brain or saliva of seropositive animals with no evidence found [51,97,98]. The implications of a carrier state for rabies epidemiology are worth consideration because even infrequent occurrence of carriers could significantly influence rabies dynamics through the exposure of large numbers of other individuals [20]. However, evidence for a carrier state is very scarce, and it is highly unlikely to account for any significant number of healthy, unvaccinated rabies-seropositive individuals.

### Extended latent period

The latent or incubation period is the interval between initial exposure to a pathogen and the onset of clinical symptoms. For rabies, the latent period varies considerably. Although in humans, this period lasts between 20 to 90 days on average [79], latent periods over a year have been reported [99]. In domestic dogs, Hampson and colleagues found an average incubation period of 22.3 days, but periods over 200 days were also recorded [76]. RVNAs might therefore indicate active infection that has not yet reached the clinical stage. However, in typical rabies infection, because of immune evasion by the virus, antibodies are not detectable during the latent period; therefore, the occurrence of an extended latent period is unlikely to explain the phenomenon of unvaccinated seropositive individuals [60]. Furthermore, there is no evidence that healthy seropositive individuals later develop clinical rabies, as would be expected if they were harbouring latent infection. For example, in Tanzania no significant difference in mortality was found between seropositive and seronegative domestic dogs, and none of the 32 dogs defined as seropositive by the RFFIT were reported to have developed clinical rabies [33]. In the wildlife studies reported, in many cases individuals were released without follow-up, making it impossible to rule out that they were incubating rabies. However, in studies that did follow up, all seropositive individuals remained healthy. It is therefore unlikely that any significant proportion of seropositive individuals detected are in the latent period.

## Implications of nonlethal infection

Whilst it remains unclear what proportion of seropositive unvaccinated individuals can be truly attributed to nonlethal rabies exposure, there is significant evidence that subclinical rabies infection does occur. This observation has potential implications for rabies epidemiology, surveillance, and control.

### Immunity to reinfection

The population-level consequences of nonlethal rabies infection depend on whether the immune response generated results in protection against future infection. The titres of RVNAs detected vary widely within populations and even in longitudinally sampled individuals. However, these titres often exceed those regarded as protective following vaccination in domestic animals. In the case of domestic dogs, challenge experiments suggest a titre of 0.2 IU/mL is sufficient for protection [100], whilst in vaccinated wildlife, a titre of >0.5 IU/mL was found to be protective in 95% of cases [32]. Even individuals with low titres because of waning antibody levels following exposure may remain protected because of the ability to develop antibodies more rapidly following secondary exposure. Furthermore, although cross-reactivity with other lyssaviruses could mean that not all RVNAs are true indicators of rabies exposure, they may still provide protection against RABV exposure [101].

Directly testing for protection would require challenging seropositive wildlife or domestic dogs with RABV. Whilst experiments of this kind may be possible, they evidently present major ethical and practical challenges. An alternative strategy to gauge the immune response of seropositive individuals to rabies exposure is to look at their response to vaccination. Initial exposure to an antigen, either through vaccination or infection, ‘primes’ the immune system; therefore, on subsequent exposure, the immune reaction is faster and stronger [102]. This heightened response is known as an anamnestic response and allows for rapid clearance of the pathogen before onset of clinical infection. Testing for this anamnestic response can provide an indication of how seropositive individuals would react to challenge. However, vaccination differs significantly from natural exposure because the antigen is highly purified and immunogenic. Therefore, a response to vaccination does not equal protection to exposure to a wild-type strain. In Haiti, measurement of RVNA before and after vaccination showed a greater increase following vaccination in dogs that were seropositive prevaccination than seronegative matched controls [103], suggesting an anamnestic response occurred in seropositive individuals. However, only 10 seropositive dogs were studied. Bahloul and colleagues also found that dogs in field conditions showed higher antibody titres postvaccination than those kept under experimental conditions, potentially due to previous subclinical infection [30]. In cattle, Gilbert and colleagues also found that prevaccination RVNA was marginally associated with a stronger postvaccination response [104]. By contrast, a study of dogs in Thailand found no evidence of an anamnestic response, with seropositive results detected by an RFFIT suggested to be false positives [105]. These studies all compared the response at between 13 days to 6 months postvaccination. Because even fully naïve individuals will have generated a response to vaccination by these time points, distinguishing between an anamnestic and naïve response is challenging. Further experiments of this kind are therefore recommended to help clarify whether seropositive individuals show an anamnestic response, as expected if they result from nonlethal exposure.

### Incorporating nonlethal infection into models of rabies dynamics

Estimates of vaccination coverage required to control rabies are based on mathematical models of the disease. Classically, rabies is modelled using SEI (susceptible, exposed, infectious) or SEIV (susceptible, exposed, infectious, vaccinated) compartmental models, with no recovered class (e.g., [106,107]). In some cases such as fox rabies, in which susceptibility is very high with little evidence of postinfection immunity, this assumption of 100% fatality may be appropriate [108]. However, in species in which high seroprevalences have been detected in a number of studies, such as bat species, domestic dogs, and mongoose species, this assumption may be invalid (see Table 2). Consideration of naturally acquired immunity through inclusion of a recovered class (susceptible, exposed, infectious, recovered [SEIR]) in models may therefore provide more accurate model estimates. However, further study is required to accurately inform parametrisation of immunity in rabies models; for example, on the proportion of individuals developing immunity and the duration of immunity. This could have implications for both our understanding of rabies dynamics and the design of control strategies.

### Role of nonlethal rabies in persistence

The high host mortality rate associated with RABV, together with its infection of slowly reproducing mammalian hosts, has led to questions as to how the virus persists within populations. Whilst the high cross-species transmissibility of the virus means it is not limited to a single host population, strains of RABV are closely associated with and adapted to specific host species [109]. If RVNAs are indicative of frequent nonlethal exposure, this could suggest that rabies occurs in a more typical host–parasite relationship with lower mortality than is often portrayed [18]. For example, in vampire bats, the occurrence of immunizing nonlethal infections, together with between-colony dispersal, was found to be necessary for the maintenance of rabies within populations [75, 110]. Although immune hosts do not transmit virus, they are still able to reproduce, therefore replenishing the susceptible population and providing the virus with new hosts. Lower virulence may therefore benefit viral persistence in smaller populations or when cross-species transmission is less frequent. If the virus is able to adapt to facilitate a carrier state in which it can be transmitted without causing symptoms, this could also favour viral persistence. Cleaveland and Dye found that in a model of rabies in dog populations, persistence was much more likely if seropositive individuals are carriers [20]. When cycles of rabies infection occur, the presence of carriers makes extinction much less likely in the ‘troughs’ between peaks of infection. As a result, if a carrier state does occur, it could be an important mechanism in long-term maintenance in a system.

### Rabies surveillance

Canine vaccination has been shown to be effective in reducing the rabies public health burden, and progress is being made towards the goal of eradicating human rabies transmitted by dogs by 2030 [111]. Oral vaccination has also been demonstrated to be a successful strategy for control of independent cycles of rabies in wildlife. However, in not all cases is it evident which species are maintaining rabies [112]. Surveillance for rabies is therefore of key importance for control efforts, both to monitor the progress of eradication in domestic dogs and to inform the focus of control efforts in wildlife. Serology provides a method of assessing pathogen exposure without the need to fatally sample or sample during active infection. However, detection of false positives remains a significant challenge, and identifying the appropriate test and cutoff to provide high specificity is central to the use of rabies serology in surveillance [113]. Interpreting disease prevalence from serology data is also limited by current understanding of rabies serological responses. Factors such as the case fatality ratio, the probability of seroconversion, and the duration of the antibody response have implications for how serology studies are interpreted [52]. Further study is required to clarify these factors, as well as the test methods most appropriate for identifying true nonlethal exposures.

## Conclusions

The occurrence of nonlethal rabies infections has been recognised since Pasteur; however, the view of rabies exposure as inevitably fatal is still widespread. A key line of evidence against this view is the detection of RVNAs in healthy, unvaccinated individuals. Whilst experimental studies support that subclinical rabies infection with subsequent immunity does occur, estimating the prevalence of this under natural conditions is complicated by the challenges of interpreting serology methods. To fully understand the role of nonlethal rabies, the following questions require further study: 1) what test methods and cutoffs are most appropriate for specific detection of rabies antibodies in naturally exposed individuals; 2) what proportion of RVNAs in healthy, unvaccinated individuals are true indicators of subclinical rabies exposure; and 3) are naturally exposed rabies-seropositive individuals immune to reinfection? Answering these questions could help to clarify rabies ecology and challenge the still widely held view of rabies exposure as inevitably fatal. This could lead to improved models for understanding rabies dynamics and designing surveillance methods.

**Key learning points**

- Rabies-specific antibodies in the sera of healthy, unvaccinated individuals have been reported in a number of studies of wildlife, domestic dogs, and humans. These antibodies provide potential evidence for nonlethal rabies exposure.
- The specificity and sensitivity of serology tests for detecting rabies exposures depends on the test method and cutoff titre chosen, which varies between studies. Together with the possibility for cross-reactivity with other lyssaviruses, this poses a significant challenge to estimating the true prevalence of nonlethal rabies exposure.
- In cases in which nonlethal rabies exposure occurs, subclinical infection, in which the virus is cleared before the onset of recognisable clinical symptoms, is the most likely alternative course of infection.
- Improved estimates of rabies seroprevalence in wildlife and domestic dogs could potentially be used to inform rabies disease models and surveillance methods.

**Key papers**

1. Bell JF. Latency and abortive rabies. In: Baer GM, editor. The natural history of rabies. New York, New York: Academic Press; 1975. pp. 331–354.
2. Carey AB, McLean RG. The ecology of rabies: evidence of co-adaptation. J Appl Ecol. 1983:20(3):777–800.
3. Cleaveland S, Dye C. Maintenance of a microparasite infecting several host species: rabies in the Serengeti. Parasitol. 1995;111(S1):S33–47.
4. Cleaveland S, Barrat J, Barrat MJ, Selve M, Kaare M, Esterhuysen J. A rabies serosurvey of domestic dogs in rural Tanzania: results of a rapid fluorescent focus inhibition test (RFFIT) and a liquid-phase blocking ELISA used in parallel. Epidemiol Infect. 1999;123(1):157–164.
5. Gilbert AT, Petersen BW, Recuenco S, Niezgoda M, Gómez J, Laguna-Torres VA, et al. Evidence of rabies virus exposure among humans in the Peruvian Amazon. Am J Trop Med Hyg. 2012;87(2):206–215.

## Supporting information

S1 Table Additional information on serology studies in unvaccinated domestic dogs. Row number of study relative to Table 1 is shown. Information is provided on whether each study followed seropositive individuals to see if they developed rabies, evidence for previous vaccination, notes on the test method used, and any additional information of use for interpreting the study results.
(PDF)

S2 Table Additional information on serology studies in unvaccinated wildlife. Row number of study relative to Table 2 is shown. Additional information of use for interpreting the reported seroprevalence is shown. This includes whether low cutoffs were used, variation in test methods, and whether follow-up was carried out to check for development of symptoms.
(PDF)

## References

[1] Walker PJ, Blasdell KR, Calisher CH, Dietzgen RG, Kondo H, Kurath G, Longdon B, Stone DM, Tesh RB, Tordo N, Vasilakis N. ICTV virus taxonomy profile: Rhabdoviridae. J Gen Virol. 2018;99(4):447–448. 10.1099/jgv.0.001020 29465028 PMC12662152

[2] Hampson K, Coudeville L, Lembo T, Sambo M, Kieffer A, Attlan M, Barrat J, Blanton JD, Briggs DJ, Cleaveland S, Costa P. Estimating the global burden of endemic canine rabies. PLoS Negl Trop Dis. 2015;9(4):e0003709 10.1371/journal.pntd.0003709 25881058 PMC4400070

[3] World Health Organisation. WHO Expert Consultation on Rabies: second report. WHO Technical Report Series 982 Geneva, Switzerland: WHO; 2013 [cited 2018 Dec 13]. Available from: https://apps.who.int/iris/bitstream/handle/10665/85346/9789241209823_eng.pdf?sequence=1. 24069724

[4] Lembo T, Hampson K, Kaare MT, Ernest E, Knobel D, Kazwala RR, Haydon DT, Cleaveland S. The feasibility of canine rabies elimination in Africa: dispelling doubts with data. PLoS Negl Trop Dis. 2010;4(2):e626 10.1371/journal.pntd.0000626 20186330 PMC2826407

[5] Coleman PG, Dye C. Immunization coverage required to prevent outbreaks of dog rabies. Vaccine. 1996;14(3):185–6. 10.1016/0264-410x(95)00197-9 8920697

[6] Fitzpatrick MC, Hampson K, Cleaveland S, Meyers LA, Townsend JP, Galvani AP. Potential for rabies control through dog vaccination in wildlife-abundant communities of Tanzania. PLoS Negl Trop Dis. 2012;6(8):e1796 10.1371/journal.pntd.0001796 22928056 PMC3424251

[7] Kitala PM, McDermott JJ, Coleman PG, Dye C. Comparison of vaccination strategies for the control of dog rabies in Machakos District, Kenya. Epidemiol Infect. 2002;129(1):215–22. 10.1017/s0950268802006957 12211590 PMC2869868

[8] Morters MK, Restif O, Hampson K, Cleaveland S, Wood JL, Conlan AJ. Evidence‐based control of canine rabies: a critical review of population density reduction. J Anim Ecol. 2013;82(1):6–14. 10.1111/j.1365-2656.2012.02033.x 23004351 PMC3579231

[9] Singh R, Singh KP, Cherian S, Saminathan M, Kapoor S, Manjunatha Reddy GB, Panda S, Dhama K. Rabies epidemiology, pathogenesis, public health concerns and advances in diagnosis and control: a comprehensive review. Vet Q. 2017;37(1):212–51. 10.1080/01652176.2017.1343516 28643547

[10] Orr PH, Rubin MR, Aoki FY. Naturally acquired serum rabies neutralizing antibody in a Canadian Inuit population. Arctic Med Res. 1988;47:699 3272719

[11] Ogunkoya AB, Beran GW, Umoh JU, Gomwalk NE, Abdulkadir IA. Serological evidence of infection of dogs and man in Nigeria by lyssaviruses (family Rhabdoviridae). Trans R Soc Trop Med Hyg. 1990;84(6):842–5. 10.1016/0035-9203(90)90103-l 2096520

[12] Follmann EH, Ritter DG, Beller M. Survey of fox trappers in northern Alaska for rabies antibody. Epidemiol Infect. 1994;113(1):137–41. 10.1017/s0950268800051554 8062870 PMC2271209

[13] Gilbert AT, Petersen BW, Recuenco S, Niezgoda M, Gómez J, Laguna-Torres VA, Rupprecht C. Evidence of rabies virus exposure among humans in the Peruvian Amazon. Am J Trop Med Hyg. 2012;87(2):206–15. 10.4269/ajtmh.2012.11-0689 22855749 PMC3414554

[14] Pasteur L, Chamberland M, Roux M, Thuillier M. Nouveaux faits pour servir a la connaissance de la rage. C R Acad Sci. 1882;95:1187.

[15] Turmelle AS, Jackson FR, Green D, McCracken GF, Rupprecht CE. Host immunity to repeated rabies virus infection in big brown bats. J Gen Virol. 2010;91(Pt 9):2360 10.1099/vir.0.020073-0 20519458 PMC3052523

[16] Gilbert AT, Fooks AR, Hayman DT, Horton DL, Müller T, Plowright R, Peel AJ, Bowen R, Wood JL, Mills J, Cunningham AA. Deciphering serology to understand the ecology of infectious diseases in wildlife. Ecohealth. 2013;10(3):298–313. 10.1007/s10393-013-0856-0 23918033

[17] Bell JF. Latency and abortive rabies In: Baer GM, editor. The natural history of rabies. New York, New York: Academic Press; 1975 pp. 331–354.

[18] Carey AB, McLean RG. The ecology of rabies: evidence of co-adaptation. J Appl Ecol. 1983;1:777–800.

[19] Fekadu M. Canine rabies. In: Baer GM, editor. The natural history of rabies: 2nd edition. Boca Raton: CRC Press; 1991 pp. 367–387.

[20] Cleaveland S, Dye C. Maintenance of a microparasite infecting several host species: rabies in the Serengeti. Parasitol. 1995;111(S1):S33–47. 10.1017/s0031182000075806 8632923

[21] Webster LT, Dawson JR Jr. Early diagnosis of rabies by mouse inoculation. Measurement of humoral immunity to rabies by mouse protection test. Proc Soc Exp Biol Med. 1935;32(4):570–3.

[22] Lord R, Fuenzalida E, Delpietro H, Larghi O, Diaz A, Lazaro L. Observations on the epizootiology of vampire bat rabies. Bull Pan Am Health Organ. 1975;82(6):498–505. 1212534

[23] Bigler WJ, Hoff GL, Smith JS, McLean RG, Trevino HA, Ingwersen J. Persistence of rabies antibody in free-ranging raccoons. J Infect Dis. 1983;148(3):610 10.1093/infdis/148.3.610 6619583

[24] OIE World Organisation for Animal Health. Rabies (Infection with rabies virus and other lyssaviruses) In: Manual of Diagnostic Tests and Vaccines for Terrestrial Animals Paris, France: OIE; 2018.

[25] Cliquet F, Aubert M, Sagne L. Development of a fluorescent antibody virus neutralisation test (FAVN test) for the quantitation of rabies-neutralising antibody. J Immunolog Methods. 1998;212(1):79–87. 10.1016/s0022-1759(97)00212-3 9671155

[26] Zalan E, Wilson C, Pukitis D. A microtest for the quantitation of rabies virus neutralizing antibodies. J Biol Standardization. 1979;7(3):213–20. 10.1016/s0092-1157(79)80024-4 387798

[27] Wright E, McNabb S, Goddard T, Horton D L, Lembo T, Nel L. H., et al A robust lentiviral pseudotype neutralisation assay for in-field serosurveillance of rabies and lyssaviruses in Africa. Vaccine. 2009;27(51):7178–86. 10.1016/j.vaccine.2009.09.024 19925950 PMC2789314

[28] Esterhuysen JJ, Prehaud C, Thomson GR. A liquid-phase blocking ELISA for the detection of antibodies to rabies virus. J Virological Methods. 1995;51(1):31–42 10.1016/0166-0934(94)00098-2 7730435

[29] Welch RJ, Anderson BL, Litwin CM. An evaluation of two commercially available ELISAs and one in-house reference laboratory ELISA for the determination of human anti-rabies virus antibodies. J Med Microbiol. 2009;58(6):806–10. 19429758 10.1099/jmm.0.006064-0

[30] Bahloul C, Taieb D, Kaabi B, Diouani MF, Hadjahmed SB, Chtourou Y, B'chir BI, Dellagi K. Comparative evaluation of specific ELISA and RFFIT antibody assays in the assessment of dog immunity against rabies. Epidemiol Infect. 2005;133(4):749–57. 10.1017/s095026880500381x 16050522 PMC2870304

[31] Wasniewski M, Cliquet F. Evaluation of ELISA for detection of rabies antibodies in domestic carnivores. J Virological Methods. 2012;179(1):166–75. 10.1016/j.jviromet.2011.10.019 22080853

[32] Moore SM, Gilbert A, Vos A, Freuling C M, Ellis C, Kliemt J, & Müller T. Rabies virus antibodies from oral vaccination as a correlate of protection against lethal infection in wildlife. Trop Med Infect Dis. 2017;2(3):31. 10.3390/tropicalmed2030031 PMC6082110 30270888

[33] Cleaveland S, Barrat J, Barrat MJ, Selve M, Kaare M, Esterhuysen J. A rabies serosurvey of domestic dogs in rural Tanzania: results of a rapid fluorescent focus inhibition test (RFFIT) and a liquid-phase blocking ELISA used in parallel. Epidemiol Infect. 1999;123(1):157–64. 10.1017/s0950268899002563 10487652 PMC2810739

[34] Cliquet F, Müller T, Mutinelli F, Geronutti S, Brochier B, Selhorst T, Schereffer JL, Krafft N, Burow J, Schameitat A, Schlüter H. Standardisation and establishment of a rabies ELISA test in European laboratories for assessing the efficacy of oral fox vaccination campaigns. Vaccine. 2003;21(21–22):2986–93. 10.1016/s0264-410x(03)00102-6 12798642

[35] Mebatsion T, Sillero‐Zubiri C, Gottelli D, Cox JH. Detection of rabies antibody by ELISA and RFFIT in unvaccinated dogs and in the endangered Simien jackal (*Canis simensis*) of Ethiopia. Zentralbl Veterinarmed B. 1992;39(1‐10):233–5. 1642078 10.1111/j.1439-0450.1992.tb01162.x

[36] Moore SM, Hanlon CA. Rabies-specific antibodies: measuring surrogates of protection against a fatal disease. PLoS Negl Trop Dis. 2010;4(3):e595 10.1371/journal.pntd.0000595 20231877 PMC2834733

[37] World Health Organisation. WHO Expert Consultation on Rabies: first report. WHO Technical Report Series 931 Geneva, Switzerland: WHO; 2004 [cited 2018 Dec 13]. Available from: https://www.who.int/rabies/ExpertConsultationOnRabies.pdf?ua=1.

[38] Greiner M, Pfeiffer D, Smith RD. Principles and practical application of the receiver-operating characteristic analysis for diagnostic tests. Prev Vet Med. 2000;45(1–2):23–41. 10.1016/s0167-5877(00)00115-x 10802332

[39] Gardner IA, Hietala S, Boyce WM. Validity of using serological tests for diagnosis of diseases in wild animals. Rev Sci Tech. 1996;15:323–332. 10.20506/rst.15.1.926 8924713

[40] Peel A J, McKinley TJ, Baker KS, Barr JA, Crameri G., Hayman DT, et al Use of cross-reactive serological assays for detecting novel pathogens in wildlife: assessing an appropriate cut off for henipavirus assays in African bats. J Virological Methods. 2013;193(2):295–303. 10.1016/j.jviromet.2013.06.030 PMC8796701 23835034

[41] Hayman DT, Fooks AR, Marston DA, Garcia-RJC. The global phylogeography of lyssaviruses-challenging the 'out of Africa' hypothesis. PLoS Negl Trop Dis. 2016;10(12):e0005266 10.1371/journal.pntd.0005266 28036390 PMC5231386

[42] Brookes SM, Parsons G, Johnson N, McElhinney LM, Fooks AR. Rabies human diploid cell vaccine elicits cross-neutralising and cross-protecting immune responses against European and Australian bat lyssaviruses. Vaccine. 2005;23(32):4101–9. 10.1016/j.vaccine.2005.03.037 15964478

[43] Wright E, Temperton NJ, Marston DA, McElhinney LM, Fooks AR, Weiss RA. Investigating antibody neutralization of lyssaviruses using lentiviral pseudotypes: a cross-species comparison. J Gen Virol. 2008;89(Pt 9):2204 10.1099/vir.0.2008/000349-0 18753230 PMC2886951

[44] Horton DL, McElhinney LM, Marston DA, Wood JLN, Russell CA, Lewis N, et al Quantifying antigenic relationships among the lyssaviruses. J Virology. 2010;84(22):11841–8. 10.1128/JVI.01153-10 20826698 PMC2977894

[45] Berentsen AR, Dunbar MR, Becker MS, M'soka J, Droge E, Sakuya NM, Matandiko W, McRobb R, Hanlon CA. Rabies, canine distemper, and canine parvovirus exposure in large carnivore communities from two Zambian ecosystems. Vector Borne Zoonotic Dis. 2013;13(9):643–9. 10.1089/vbz.2012.1233 23805791

[46] Wright E, Temperton NJ, Marston DA, McElhinney LM, Fooks AR, Weiss RA. Investigating antibody neutralization of lyssaviruses using lentiviral pseudotypes: a cross-species comparison. J Gen Virol. 2008;89(Pt 9):2204 10.1099/vir.0.2008/000349-0 18753230 PMC2886951

[47] Mélade J, McCulloch S, Ramasindrazana B, Lagadec E, Turpin M, Pascalis H, Goodman SM, Markotter W, Dellagi K. Serological evidence of lyssaviruses among bats on Southwestern Indian Ocean Islands. PLoS ONE. 2016 8 8;11(8):e0160553 10.1371/journal.pone.0160553 27501458 PMC4976896

[48] Müller T, Selhorst T, Burow J, Schameitat A, Vos A. Cross reactive antigenicity in orally vaccinated foxes and raccoon dogs against European Bat Lyssavirus type 1 and 2. Dev Biol. 2006;125:195–204. 16878477

[49] Rudd RJ, Appler KA, Wong SJ. Presence of cross-reactions with other viral encephalitides in the indirect fluorescent-antibody test for diagnosis of rabies. J Clin Microbiol. 2013;51(12):4079–82. 10.1128/JCM.01818-13 24088851 PMC3838086

[50] Kostense S, Moore S, Companjen A, Bakker AB, Marissen WE, von Eyben R, et al Validation of the rapid fluorescent focus inhibition test for rabies virus-neutralizing antibodies in clinical samples. Antimicrob Agents Chemother. 2012;56(7):3524–30. 10.1128/AAC.06179-11 22547629 PMC3393455

[51] Prager KC, Mazet JA, Dubovi EJ, Frank LG, Munson L, Wagner AP, et al Rabies virus and canine distemper virus in wild and domestic carnivores in Northern Kenya: Are domestic dogs the reservoir? EcoHealth. 2012;9(4):483–98. 10.1007/s10393-013-0815-9 23459924

[52] Pepin KM, Kay SL, Golas BD, Shriner SS, Gilbert AT, Miller RS, et al Inferring infection hazard in wildlife populations by linking data across individual and population scales. Ecol Lett. 2017;20(3):275–92. 10.1111/ele.12732 28090753 PMC7163542

[53] Laurenson K, Van Heerden J, Stander P, Van Vuuren MJ. Seroepidemiological survey of sympatric domestic and wild dogs (*Lycaon pictus*) in Tsumkwe District, northeastern Namibia. Onderstepoort J Vet Res. 1997;64:313–316. 9551484

[54] Gascoyne SC, King AA, Laurenson MK, Borner M, Schildger B, Barrat J. Aspects of rabies infection and control in the conservation of the African wild dog (*Lycaon pictus*) in the Serengeti region, Tanzania. Onderstepoort J Vet Res. 1993;60:415 7777330

[55] Deem SL, Davis R, Pacheco LF. Serologic evidence of nonfatal rabies exposure in a free-ranging oncilla (*Leopardus tigrinus*) in Cotapata National Park, Bolivia. J Wildl Dis. 2004;40(4):811–5. 10.7589/0090-3558-40.4.811 15650107

[56] Ramey PC, Blackwell BF, Gates RJ, Slemons RD. Oral rabies vaccination of a northern Ohio raccoon population: relevance of population density and prebait serology. J Wildl Dis. 2008;44(3):553–68. 10.7589/0090-3558-44.3.553 18689640

[57] Araujo DB, Martorelli LA, Kataoka AP, Campos AC, Rodrigues CS, Sanfilippo LF, et al Antibodies to rabies virus in terrestrial wild mammals in native rainforest on the north coast of São Paulo State, Brazil. J Wildl Dis. 2014;50(3):469–77. 10.7589/2013-04-099 24779464

[58] Machado GP, de Paula Antunes JMA, Uieda W, Biondo AW, de Andrade Cruvinel TM, Kataoka AP, et al Exposure to rabies virus in a population of free-ranging capuchin monkeys (*Cebus apella nigritus*) in a fragmented, environmentally protected area in southeastern Brazil. Primates. 2012;53(3):227–231. 10.1007/s10329-012-0306-6 22430558

[59] McLean RG. Rabies in raccoons in the Southeastern United States. J Infect Dis. 1971;123(6):680–81. 10.1093/infdis/123.6.680 5165034

[60] Johnson N, Cunningham AF, Fooks AR. The immune response to rabies virus infection and vaccination. Vaccine. 2010;28(23):3896–901. 10.1016/j.vaccine.2010.03.039 20368119

[61] Petersen BW, Rupprecht CE. Human rabies epidemiology and diagnosis In: Tkachev S, editor. Non-Flavivirus encephalitis. Rijeka, Croatia: In Tech; 2011 pp. 247–278.

[62] Lafon M. Immune evasion, a critical strategy for rabies virus. Dev Biol. 2008;131:413–419. 18634503

[63] Gnanadurai CW, Zhou M, He W, Leyson CM, Huang CT, Salyards G, et al Presence of virus neutralizing antibodies in cerebral spinal fluid correlates with non-lethal rabies in dogs. PLoS Negl Trop Dis. 2013;7(9):e2375 10.1371/journal.pntd.0002375 24069466 PMC3777866

[64] Manickam R, Basheer MD, Jayakumar R. Post-exposure prophylaxis (PEP) of rabies-infected Indian street dogs. Vaccine. 2008;26(51):6564–8. 10.1016/j.vaccine.2008.09.053 18848596

[65] Bell JF. Abortive rabies infection: I. Experimental production in white mice and general discussion. J Infect Dis. 1964:249–57. 10.1093/infdis/114.3.249 14183397

[66] Lodmell DL, Bell JF, Moore GJ, Raymond GH. Comparative study of abortive and nonabortive rabies in mice. J Infect Dis. 1969;119(6):569–80. 10.1093/infdis/119.6.569 4893889

[67] McColl KA, Tordo N, Aguilar Setien A. Bat lyssavirus infections. Rev Sci Tech. 2000;19(1):177–189. 10.20506/rst.19.1.1221 11189715

[68] Almeida MFD, Martorelli LFA, Aires CC, Sallum PC, Durigon EL, Massad E. Experimental rabies infection in haematophagous bats *Desmodus rotundus*. Epidemiol Infec. 2005;133(3): 523–527. 15962559 10.1017/s0950268804003656 PMC2870276

[69] Jackson FR, Turmelle AS, Farino DM, Franka R, McCracken GF, Rupprecht CE. Experimental rabies virus infection of big brown bats (*Eptesicus fuscus*). J Wildlife Dis. 2008;44(3):612–21. 10.7589/0090-3558-44.3.612 18689646

[70] Obregón-Morales C, Aguilar-Setién Á, Martínez LP, Galvez-Romero G, Martínez-Martínez FO, Aréchiga-Ceballos N. Experimental infection of *Artibeus intermedius* with a vampire bat rabies virus. Comp Immunol Microbiol Infect Dis. 2017;52:43–47. 10.1016/j.cimid.2017.05.008 28673461

[71] Calisher CH, Childs JE, Field HE, Holmes KV, Schountz T. Bats: important reservoir hosts of emerging viruses. Clin Microbiol Rev. 2006;19(3):531–45. 10.1128/CMR.00017-06 16847084 PMC1539106

[72] Rupprecht C, Kuzmin I, Meslin F. Lyssaviruses and rabies: current conundrums, concerns, contradictions and controversies. F1000Res. 2017;6:184 10.12688/f1000research.10416.1 28299201 PMC5325067

[73] Jorge RS, Pereira MS, Morato RG, Scheffer KC, Carnieli P Jr, Ferreira F, et al Detection of rabies virus antibodies in Brazilian free-ranging wild carnivores. J Wildl Dis. 2010 10;46(4):1310–1315. 10.7589/0090-3558-46.4.1310 20966286

[74] Bellan SE, Cizauskas CA, Miyen J, Ebersohn K, Küsters M, Prager KC, et al Black-backed jackal exposure to rabies virus, canine distemper virus, and *Bacillus anthracis* in Etosha National Park, Namibia. J Wildl Dis. 2012;48(2):371–81. 10.7589/0090-3558-48.2.371 22493112 PMC5479413

[75] Blackwood JC, Streicker DG, Altizer S, Rohani P. Resolving the roles of immunity, pathogenesis, and immigration for rabies persistence in vampire bats. Proc Natl Acad Sci USA. 2013;110(51):20837–42. 10.1073/pnas.1308817110 24297874 PMC3870737

[76] Hampson K, Dushoff J, Cleaveland S, Haydon DT, Kaare M, Packer C, Dobson A. Transmission dynamics and prospects for the elimination of canine rabies. PLoS Biol. 2009 3 10;7(3):e1000053. 10.1371/journal.pbio.1000053 PMC2653555 19278295

[77] Changalucha J, Steenson R, Grieve E, Cleaveland S, Lembo T, Lushasi K, et al The need to improve access to rabies post-exposure vaccines: lessons from Tanzania. Vaccine. 2019;37:A45–53. 10.1016/j.vaccine.2018.08.086 30309746 PMC6863039

[78] de Souza A, Madhusudana SN. Survival from rabies encephalitis. J Neurological Sci. 2014;339(1–2):8–14. 10.1016/j.jns.2014.02.013 24582283

[79] Jackson AC, Warrell MJ, Rupprecht CE, Ertl HC, Dietzschold B, O'Reilly M, et al Management of rabies in humans. Clin Infect Dis. 2003 1 1;36(1):60–63. 10.1086/344905 12491203

[80] Jackson AC, Reimer DL, Ludwin SK. Spontaneous recovery from the encephalomyelitis in mice caused by street rabies virus. Neuropathol Appl Neurobiol. 1989;15(5):459–75. 10.1111/j.1365-2990.1989.tb01246.x 2586721

[81] Fekadu M, Baer GM. Recovery from clinical rabies of 2 dogs inoculated with a rabies virus strain from Ethiopia. Am J Vet Res. 1980;41(10):1632–4. 7224288

[82] Hamir AN, Niezgoda M, Rupprecht CE. Recovery from and clearance of rabies virus in a domestic ferret. J Am Assoc Lab Anim Sci. 2011;50(2):248–51. 21439220 PMC3061427

[83] Gribencha SV. Abortive rabies in rabbits and white rats infected intracerebrally. Arch Virol. 1975;49(4):317–22. 10.1007/bf01318240 1212100

[84] Roy A, Phares TW, Koprowski H, Hooper DC. Failure to open the blood-brain barrier and deliver immune effectors to central nervous system tissues leads to the lethal outcome of silver-haired bat rabies virus infection. J Virol. 2007;81(3):1110–8. 10.1128/JVI.01964-06 17108029 PMC1797506

[85] Hooper DC, Roy A, Barkhouse DA, Li J, Kean RB. Rabies virus clearance from the central nervous system. Adv Virus Res. 2011;79:55–71. 10.1016/B978-0-12-387040-7.00004-4 21601042

[86] Lodmell DL, Ewalt LC. Pathogenesis of street rabies virus infections in resistant and susceptible strains of mice. J Virol. 1985;55(3):788–95. 4020967 10.1128/jvi.55.3.788-795.1985 PMC255063

[87] Bell JF, Lodmell DL, Moore GJ, Raymond GH. Brain neutralization of rabies virus to distinguish recovered animals from previously vaccinated animals. J Immunol. 1966;97(6):747–53. 5955831

[88] Tepsumethanon V, Lumlertdacha B, Mitmoonpitak C, Sitprija V, Meslin FX, Wilde H. Survival of naturally infected rabid dogs and cats. Clin Infect Dis. 2004;39(2):278–80. 10.1086/421556 15307040

[89] Pawan JL. Rabies in the vampire bat of Trinidad, with special reference to the clinical course and the latency of infection. Ann Trop Med Parasitol. 1936;30(4):401–22. 14431118

[90] Aguilar-Setien A, Loza-Rubio E, Salas-Rojas M, Brisseau N, Cliquet F, Pastoret PP, Rojas-Dotor S, Tesoro E, Kretschmer R. Salivary excretion of rabies virus by healthy vampire bats. Epidemiol Infect. 2005;133(3):517–22. 10.1017/s0950268805003705 15966107 PMC2870282

[91] Moreno JA, Baer GM. Experimental rabies in the vampire bat. Am J Trop Med Hyg. 1980;29(2):254–9. 10.4269/ajtmh.1980.29.254 7369444

[92] Fekadu M, Shaddock JH, Chandler FW, Baer GM. Rabies virus in the tonsils of a carrier dog. Arch Virol. 1983;78(1–2):37–47. 10.1007/bf01310857 6651535

[93] Aghomo HO, Rupprecht CE. Further studies on rabies virus isolated from healthy dogs in Nigeria. Vet Microbiol. 1990;22(1):17–22. 10.1016/0378-1135(90)90120-k 2186564

[94] Zhang YZ, Fu ZF, Wang DM, Zhou JZ, Wang ZX, Lv TF, Xiong CL, Zou Y, Yao WR, Li MH, Dong GM. Investigation of the role of healthy dogs as potential carriers of rabies virus. Vector Borne Zoonotic Dis. 2008;8(3):313–20. 10.1089/vbz.2007.0209 18380590

[95] Bell JF, Gonzalez MA, Diaz AM, Moore GJ. Nonfatal rabies in dogs: experimental studies and results of a survey. Amer J Vet Res. 1971;32:2049–2058. 4943410

[96] Raju S, Saseendranath MR. Screening of dogs for rabies virus excretion. J Vet Anim Sci. 2009;40:40–41.

[97] Ballard WB, Follmann EH, Ritter DG, Robards MD, Cronin MA. Rabies and canine distemper in an arctic fox population in Alaska. J Wildl Dis. 2001;37(1):133–7. 10.7589/0090-3558-37.1.133 11272487

[98] Berentsen AR, Johnson SR, Gilbert AT, VerCauteren KC. Exposure to rabies in small Indian mongooses (*Herpestes auropunctatus*) from two regions in Puerto Rico. J Wildl Dis. 2015;51(4):896–900. 10.7589/2015-01-016 26251987

[99] Smith JS, Fishbein DB, Rupprecht CE, Clark K. Unexplained Rabies in Three Immigrants in the United States a Virologic Investigation. N Engl J Med. 1991;324(4):205–11. 10.1056/NEJM199101243240401 1985241

[100] Aubert MF. Practical significance of rabies antibodies in cats and dogs. Rev Sci Tech. 1992;11:735 10.20506/rst.11.3.622 1472723

[101] Bahloul C, Jacob Y, Tordo N, Perrin P. DNA-based immunization for exploring the enlargement of immunological cross-reactivity against the lyssaviruses. Vaccine. 1998;16(4):417–25. 10.1016/s0264-410x(97)00204-1 9607065

[102] Derbyshire JB, Mathews KA. Rabies antibody titres in vaccinated dogs. Can Vet J. 1984;25(10):383 17422460 PMC1790664

[103] Smith TG, Millien M, Vos A, Fracciterne FA, Crowdis K, Chirodea C, et al Evaluation of immune responses in dogs to oral rabies vaccine under field conditions. Vaccine. 2019;37(33):4743–4749. Epub 2017 Oct 17. 10.1016/j.vaccine.2017.09.096 29054727 PMC5904025

[104] Gilbert A, Greenberg L, Moran D, Alvarez D, Alvarado M, Garcia DL, Peruski L. Antibody response of cattle to vaccination with commercial modified live rabies vaccines in Guatemala. Prev Vet Med. 2015;118(1):36–44. 10.1016/j.prevetmed.2014.10.011 25466762

[105] Tepsumethanon W, Polsuwan C, Lumlertdaecha B, Khawplod P, Hemachudha T, Chutivongse S, et al Immune response to rabies vaccine in Thai dogs: a preliminary report. Vaccine. 1991;9(9):627–30. 10.1016/0264-410x(91)90186-a 1950096

[106] Anderson RM, Jackson HC, May RM, Smith AM. Population dynamics of fox rabies in Europe. Nature. 1981;289(5800):765 10.1038/289765a0 7464941

[107] Bilinski AM, Fitzpatrick MC, Rupprecht CE, Paltiel AD, Galvani AP. Optimal frequency of rabies vaccination campaigns in sub-Saharan Africa. Proc R Soc Lond B Biol Sci. 2016;283(1842):20161211. 10.1098/rspb.2016.1211 PMC5124087 27852799

[108] Blancou J. Ecology and epidemiology of fox rabies. Rev Infect Dis. 1988;10(4):S606–9. 3060953 10.1093/clinids/10.supplement_4.s606

[109] Fisher CR, Streicker DG, Schnell MJ. The spread and evolution of rabies virus: conquering new frontiers. Nat Rev Microbiol. 2018;16(4):241 10.1038/nrmicro.2018.11 29479072 PMC6899062

[110] Streicker DG, Winternitz JC, Satterfield DA, Condori-Condori RE, Broos A, Tello C, et al Host–pathogen evolutionary signatures reveal dynamics and future invasions of vampire bat rabies. Proc Natl Acad Sci. 2016;113(39):10926–31. 10.1073/pnas.1606587113 27621441 PMC5047211

[111] World Health Organization. Zero by 30: The Global Strategic Plan to Prevent Human Deaths from Dog-Transmitted Rabies by 2030 Geneva, Switzerland: WHO; 2015 [cited 2018 Dec 13]. Available from: https://www.who.int/rabies/Executive_summary_draft_V3_wlogo.pdf?ua=1.

[112] Haydon DT, Cleaveland S, Taylor LH, Laurenson MK. Identifying reservoirs of infection: a conceptual and practical challenge. Emerg Infect Dis. 2002;8(12):1468–73. 10.3201/eid0812.010317 12498665 PMC2738515

[113] Guberti V, Stancampiano L, Ferrari N. Surveillance, monitoring and survey of wildlife diseases: a public health and conservation approach. Italian J Mammology. 2014;25(1):3–8.

[114] Wosu LO, Anyanwu HN. Seroepidemiological survey of rabies virus antibodies in non vaccinated dogs in Nsukka environs, Nigeria. J Vet Med. Series B. 1990;37(1‐10):47–52. 10.1111/j.1439-0450.1990.tb01025.x 2346070

[115] Alexander KA, Smith JS, Macharia MJ, King AA. Rabies in the Masai Mara, Kenya: preliminary report. Onderstepoort J Vet Res. 1993;60:411 7777329

[116] Millán J, Chirife AD, Kalema-Zikusoka G, Cabezón O, Muro J, Marco I, Cliquet F, León-Vizcaíno L, Wasniewski M, Almería S, Mugisha L. Serosurvey of dogs for human, livestock, and wildlife pathogens, Uganda. Emerg Infect Dis. 2013;19(4):680 10.3201/eid1904.121143 23750507 PMC3647413

[117] Alexander KA, Kat PW, Wayne RK, Fuller TK. Serologic survey of selected canine pathogens among free-ranging jackals in Kenya. J Wildl Dis. 1994;30(4):486–91. 10.7589/0090-3558-30.4.486 7760476

[118] Zarnke RL, Ballard WB. Serologic survey for selected microbial pathogens of wolves in Alaska, 1975–1982. J Wildl Dis. 1987;23(1):77–85. 10.7589/0090-3558-23.1.77 3029442

[119] East ML, Hofer H, Cox JH, Wulle U, Wiik H, Pitra C. Regular exposure to rabies virus and lack of symptomatic disease in Serengeti spotted hyenas. Proc Natl Acad Sci USA. 2001;98(26):15026–31. 10.1073/pnas.261411898 11742089 PMC64977

[120] Everard CO, Baer GM, James A. Epidemiology of mongoose rabies in Grenada. J Wildl Dis. 1974;10(3):190–6. 10.7589/0090-3558-10.3.190 4602354

[121] Langoni H, Souza LC, Zetun CB, Silva TC, Hoffmann JL, Silva RC. Serological survey for rabies in serum samples from vampire bats (*Desmodus rotundus*) in Botucatu region, SP, Brazil. J Venom Anim Toxins Incl Trop Dis. 2008;14(4):651–9.

[122] Trimarchi CV, Debbie JG. Naturally occurring rabies virus and neutralizing antibody in two species of insectivorous bats of New York State. J Wildl Dis. 1977;13(4):366–9. 10.7589/0090-3558-13.4.366 24228955

[123] Steece R, Altenbach JS. Prevalence of rabies specific antibodies in the Mexican free-tailed bat (*Tadarida brasiliensis mexicana*) at Lava Cave, New Mexico. J Wildl Dis. 1989;25(4):490–6. 10.7589/0090-3558-25.4.490 2681843

[124] Bronson E, Spiker H, Driscoll CP. Serosurvey for selected pathogens in free-ranging American black bears (*Ursus americanus*) in Maryland, USA. J Wildl Dis. 2014 10;50(4):829–36. 10.7589/2013-07-155 25075540

[125] Rosatte RC, Gunson JR. Presence of neutralizing antibodies to rabies virus in striped skunks from areas free of skunk rabies in Alberta. J Wildl Dis. 1984 7;20(3):171–176. 10.7589/0090-3558-20.3.171 6492318

