# Supplementary material for: Rabies virus-neutralising antibodies in healthy, unvaccinated individuals: What do they mean for rabies epidemiology?
Source: PLoS Negl Trop Dis. 2020 Feb 13;14(2):e0007933. doi: 10.1371/journal.pntd.0007933 (PMC7017994; doi:10.1371/journal.pntd.0007933)
Supplement: S1 Table — Row number of study relative to Table 1 is shown. Information is provided on whether each study followed seropositive individuals to see if they developed rabies, evidence for previous vaccination, notes on the test method used, and any additional information of use for interpreting the study results. (PDF) [file pntd.0007933.s001.pdf]

Row	Notes	Ref
1	**Follow up:** No follow up to see if symptoms developed reported.<br>**Vaccination:** Dogs all owned and no anti-rabies campaign for 5 years in area, therefore unlikely previously vaccinated.<br>**Test method:** Test method used not well-established.<br>**Further notes:** Study also included dogs less than 3 months old, therefore potential detection of maternal antibody, however above 6 months, 22.8% of dogs still defined as seropositive. 4 dogs with very high titres in the range of 1:512 to 1:1024.	[115]
2	**Follow up:** No follow up to see if symptoms developed reported.<br>**Vaccination:** Owners asked about vaccination status but potential for mistakes.<br>**Test method:** Good correlation between two tests used: MNT and RFFIT. Conducted RFFIT at two institutions and had 97% concordance. 3 dogs had high titres at 1:256.<br>**Further notes:** Tested samples for neutralisation of Lagos and Mokola virus and found seropositives but no evidence of cross-reactivity.	[11]
3	**Follow up:** No follow up to see if symptoms developed reported<br>**Vaccination:** Reported that no rabies vaccine given in this area, therefore unlikely previously vaccinated.<br>**Test method:** Three test methods used: RFFIT, indirect ELISA using protein A and biotin/avidin ELISA. Strong agreement (100%) between both ELISAs and RFFIT for defining seropositives, however gave different titres..<br>**Further notes:** Very high seroprevalence reported but small sample size.	[35]
4	**Follow up:** No follow up to see if symptoms developed reported.<br>**Vaccination:** Vaccination histories unknown but population almost entirely unvaccinated. T<br>**Test method:** Modified version of RFFIT	[116]
5	**Follow up:** No follow up to see if symptoms developed reported.<br>**Vaccination:** No access to rabies vaccination within the area.<br>**Test method:** LPBE used which may be more specific for detecting non-lethal exposure than RFFIT. No conversion to international units by comparison to a standard.<br>**Further notes:** Found clustering of seropositives which may be a result of waves of infection.	[53]
6	**Follow up:** No follow up to see if symptoms developed reported.<br>**Vaccination:** Owned dogs, therefore assumed owner knows vaccination status.<br>**Test method:** Poor agreement between RFFIT and ELISA. Used negative control from rabies-free island which showed ELISA more specific for unvaccinated individuals.<br>**Further notes:** Location of seropositives detected by ELISA correlated with location of known rabies cases.	[33]
7	**Follow up:** Vaccinated following initial sampling and re-sampled at a number of time points to look at antibody response. Higher titres both by RFFIT and ELISA in dogs kept in field conditions than those vaccinated under experimental conditions<br>**Vaccintion:** Population thought to be unvaccinated for at least 3 years but potential for older dogs to have residual antibodies. 18% seroprevalence in dogs younger than 2 years, which are unlikely to have been vaccinated.<br>**Test method:** Poor agreement between tests with higher seroprevalence detected by RFFIT than ELISA. :	[30]
8	**Follow up:** Dogs initially identified as having rabies virus antigen in saliva. Followed for 6 months then euthanized and tested for rabies virus. No clinical signs of rabies observed and no rabies virus detected.<br>**Vaccination:** Not owned, but not thought to be vaccinated.<br>**Further notes:** Titre range not reported but both described as close to 0.5IU/mL. No rabies virus RNA detected in saliva following initial antigen detection suggesting not carrier state. Small sample size and only 2 dogs reported seropositive.	[94]
9	**Follow up:** No follow up to see if symptoms developed reported.<br>**Vaccination:** Owners interviewed for history of vaccination. <4.5% of domestic dogs vaccinated in area therefore unlikely that vaccination accounts for seroprevalence.<br>**Further notes:** Low cut-off but titres >0.5 IU/mL also reported.	[51]
10	**Follow up:** No follow up to see if symptoms developed reported.<br>**Vaccination:** No previous vaccination in area apart from by authors. Vaccinated dogs excluded from estimate.<br>**Further notes:** Low cut-off and range of titres not reported.	[117]
