# Supplementary material for: Rabies virus-neutralising antibodies in healthy, unvaccinated individuals: What do they mean for rabies epidemiology?
Source: PLoS Negl Trop Dis. 2020 Feb 13;14(2):e0007933. doi: 10.1371/journal.pntd.0007933 (PMC7017994; doi:10.1371/journal.pntd.0007933)
Supplement: S2 Table — Row number of study relative to Table 2 is shown. Additional information of use for interpreting the reported seroprevalence is shown. This includes whether low cutoffs were used, variation in test methods, and whether follow-up was carried out to check for development of symptoms. (PDF) [file pntd.0007933.s002.pdf]

Row	Notes	Ref
1	Small sample size and only one seropositive individual detected. Titre not reported but >0.5 IU/mL. No follow up to look for development of symptoms reported.	[116]
2	Low cut-off used and only one individual seropositive with titre <0.5 IU/mL. 47 jackals (*Canis mesomelas)* tested for rabies RNA in saliva with no evidence found. No follow up to look for development of symptoms reported.	[51]
3	Titre range not reported and no conversion to international units by comparison to a standard. 3/7 seropositive jackals (*Canis mesomelas)* followed and remained healthy for at least 1 year.	[74]
4	Small sample size. No seropositives in pre-vaccination sera in zoo used as control (n=4). Animals followed and survived for between 5 months and 2.5 years	[54]
5	Low cut-off used. No titres above 0.5 IU/mL reported. No follow up to look for development of symptoms reported.	[51]
6	Only one seropositive detected and titre not reported. No conversion to international units by comparison to a standard. No follow up to look for development of symptoms reported.	[119]
7	Small sample size. Strong agreement (100%) between two different ELISAs and RFFIT but gave different titres. Oral vaccination has been used in this species but reported that no rabies vaccine given in this area. No follow up to look for development of symptoms reported.	[35]
8	All individuals were killed and tested for rabies in brain tissue using direct fluorescent antibody test, none found. No conversion to international units by comparison to a standard.	[97]
9	Low cut-off used. All seropositive individuals had titres <0.5 IU/mL. No follow to look for development of symptoms reported.	[73]
10	Only one individual tested but had high titre. No follow up to look for development of symptoms reported.	[73]
11	Low cut-off used. No individuals with titres higher than 0.5 IU/mL detected. Monitored for periods of 6-51 months with no development of rabies.	[73]
12	Reported presence of virus in saliva, however subsequent reports suggest this may have resulted from contamination. Individual titres not reported but high titres, >1.5 IU/mL detected. Survivorship analysis showed no effect of seropositivity on longevity.	[120]
13	Low cut-off used. No individuals with titres >0.5 IU/mL reported. No follow to look for development of symptoms reported.	[51]
14	No oral swabs positive for rabies RNA. Low cut-off used but high titres also reported. Some individuals re-captured. Recaptures showed evidence of seroconversion and change in titre.	[98]
15	Low cut-off used however 9 individuals had titres greater than 1:1000. Killed and tested for rabies virus in brain tissue by fluorescent antibody test, of 13 animals confirmed rabies positive, only 1 was seropositive with a titre of 1:11. No conversion to international units by comparison to a standard.	[121]
16	Only one seropositive detected and titre was <0.5 IU/mL. No follow to look for development of symptoms reported.	[73]
17	Small sample size. Low cut-off used and no individuals with titres >0.5 IU/mL. No follow to look for development of symptoms reported.	[73]
18	Small sample size. Low cut-off used and no individuals with titres >0.5 IU/mL. Both positive individuals followed for 15 and 24 months with no development of clinical signs	[73]
19	Only one seropositive individual reported and titre <0.5 IU/mL. No follow up to look for development of symptoms reported.	[73]
20	Small sample size. Low cut-off used, however 5 of the 8 seropositives had titres >0.5 IU/mL. All remained healthy for several years following sampling	[45]
21	Only one individual tested, however had very high titre. 5 individuals from zoos tested as control, all with antibody titres <0.05 IU/mL. Tracked for 18 months and remained healthy.	[55]
22	Increase in seroprevalence post-outbreak in cattle supports true rabies exposure. Seroprevalences shown are pre, during and post-outbreak. Titre range not reported. Killed and tissues inoculated into mice for virus isolation. No virus detected. No conversion to international units by comparison to a standard.	[22]
23	Domestic dog serum positive and negative controls used. Used ROC technique for cut-off. Tested initially using ELISA, and of those with titre >0.3 tested using RFFIT. 10.8% had titres >0.3 ELISA. Killed and tested for rabies virus by direct immunofluorescence. No positive individuals detected	[122]
24	Titre range not reported. No follow up to look for development of symptoms reported. No conversion to international units by comparison to a standard.	[123]
25	Titre range not reported. No follow to look for development of symptoms reported. No conversion to international units by comparison to a standard.	[123]
26	Very high seroprevalence reported. Used human positive and negative serum controls. Looked at IgM, which occurs shortly after exposure, as well as total RVNAs. Much lower prevalence of IgM. Titre range not reported. Killed and tested for virus in brain tissue by fluorescent rabies antibody test. No conversion to international units by comparison to a standard.	[124]
27	Low cut-off used but high titres reported. One individual had history of post-exposure prophylaxis (PEP) and two others unsure of vaccination history. PEP had titre of 0.1. No report of subsequent infection	[13]
28	No record of vaccination. 3 sera positive at 1:64. No report of subsequent infection. No conversion to international units by comparison to a standard.	[11]
29	Only one seropositive individual reported and titre <0.5 IU/mL. No follow up to look for development of symptoms reported.	[57]
30	Low cut-off used but high titres also reported. No follow to look for development of symptoms reported.	[58]
31	High titres reported. No follow up to look for development of symptoms reported. No conversion to international units by comparison to a standard.	[125]
32	Low cut-off used but high titres also reported. No follow up to look for development of symptoms reported.	[61]
33	Low cut-off used but high titres also reported. Repeats of assay showed high variation with some changing from negative to positive. Killed and brain tested for rabies virus by fluorescent antibody techniques. No seropositive individuals tested positive.	[126]
34	Low cut-off used and no individuals with titres >0.5 IU/mL. Some individuals radio-tracked and survived but not stated whether these were seropositive.	[56]
35	High seroprevalence reported. Low cut-off used but high titres also reported. RFFIT detected significantly more seropositive animals than MNT. Some individuals trapped multiple times and only titres from individuals repeat caught reported. Showed persistence of RVNA for at least 37 months. No conversion to international units by comparison to a standard.	[23]
36	Only one individual reported seropositive and titre <0.5 IU/mL. No follow up to look for development of symptoms reported.	[73]
37	Low cut-off used and neither individual had a titre >0.5 IU/mL. Positive and negative controls and dog OIE-reference serum used. No follow up to look for development of symptoms reported.	[57]
